# Pheno-App 2.0 – a mobile app for collecting phenotypic data in plant research

**DOI:** 10.1515/jib-2025-0057

**Published:** 2026-05-18

**Authors:** Annedore Söchting, Danuta Schüler, Theodor Strauch, Toni Schreiber

**Affiliations:** Leibniz Institute of Plant Genetics and Crop Plant Research (IPK) Gatersleben, Corrensstr. 3, 06466 Seeland, Germany; Data Processing Department, Julius Kühn Institute (JKI) – Federal Research Centre for Cultivated Plants, Erwin-Baur-Str. 27, 06484 Quedlinburg, Germany

**Keywords:** Android app, plant phenotyping, digital data acquisition, genebank

## Abstract

Documenting plant phenotypic data in the field is a common task necessary for the preservation of crop plant biodiversity in genebanks. Today, mobile applications are used to record data more efficiently and avoid errors. The Pheno-App is an open-source Android application developed to facilitate digital recording of plant phenotypic data at the Julius Kühn Institute (JKI) in Quedlinburg. Designed for tablets and smartphones, the application features customizable trials based on Excel formats, allowing both manual editing and machine processing. One major challenge in collecting phenotypic data during regeneration of plant genetic resources (PGR) in a genebank such as IPK is the diversity of crops and the many traits to record. To make the Pheno-App usable for specific genebank work, corresponding requirements were collected and implemented. The latest version, Pheno-App 2.0, is being developed at the IPK. Major advances over the original version include the ability to apply crop-specific trait sets within a single trial and efficiently input multiple observations per time point, such as percentage characterisations for population traits. These developments make Pheno-App 2.0 a versatile, evolving tool for genebank experts in conservation and characterisation of PGRs.

## 1 Introduction

Genebanks preserve and maintain a wide variety of crop plants and their wild relatives worldwide, thereby making a major contribution to the conservation of our food resources [1]. On the one hand, the genetic diversity of the preserved accessions can contribute to the adaptation of high-yielding varieties to rapidly changing climatic conditions [2]; on the other hand, it forms the basis for plant research. Data on plant genetic resources (PGR) plays an important role in the development and utilisation of the material [3]. In addition to passport data, a large amount of phenotypic data, i.e. data on observable traits of the plants, is collected and stored for all samples. In conjunction with genomic data, valuable information can be obtained for research and breeding [4]. Worldwide, over 5.9 million accessions are preserved *ex situ*, i.e. outside their natural habitat, in genebanks [5].

The genebank of the Leibniz Institute of Plant Genetics and Crop Plant Research (IPK) preserves around 152,000 accessions from 3,000 species [6]. These include cultivated varieties, landraces, populations, crop wild relatives and also research material. Around 8,000 samples are regenerated each year and each time phenotypic data is collected. One challenge in material regeneration in genebanks is precisely dealing with this diversity of plant genetic resources. Phenotypic data is differentiated into characterisation data and evaluation data: characterisation data refers to the recording of highly heritable traits, while evaluation data refers to traits whose expression is usually dependent on the environment [7]. The main tasks of genebank work are taxonomic determination, documentation and regeneration of PGRs. Preserving the material as it is, regeneration is performed as infrequently as possible, due to environmental adoption of plants. To ensure purity of the PGRs, the characterisation data from the oldest observations are referred to as reference values, and used for comparison of regenerated material. Reference values also allow to select suitable material for research or breeding purposes. Another challenge in performing the task of documentation is the characterisation of heterogeneous material, e.g. populations, landraces or crop wild relatives. Here, traits within the accession can occur proportionally with different values, in contrast to cultivated varieties, where traits are homogeneous within the accession. The aim of data collection is to describe the accessions as comprehensively and in as much detail as possible. Due to the number and diversity of accessions preserved in genebanks, this requires both experience and the use of effective tools. Tool-supported phenotyping using specially adapted applications that can be used on mobile devices is particularly well suited for use in genebanks. Such applications offer similar flexibility and effectiveness to paper-based recording, e.g. in the form of scoring sheets, while improving data quality through validation during recording. Currently available scoring applications are a good option for data collection in research projects and offer a variety of helpful functions, such as barcode scanning or recording of geocoordinates. However, phenotypic data needs to be documented more detailed to meet the requirements of genebanks. In this respect, the known tools lack input options.

Since 2006, phenotypic data at the IPK genebank has been recorded using a mobile Java application (GBIS/B) [7], which was finally replaced by the Android-based Pheno-App in spring 2023 to enable the use of new hardware and benefit of functions that come with the Android operating system and software libraries. The Pheno-App was developed at the Julius Kühn Institute in Quedlinburg and released in 2022 (version 1.0) as open source code under the Apache 2.0 licence [8]. Based on the source code of version 1.0, the Pheno-App was further developed at IPK, taking into account the requirements for recording phenotypic data at genebanks. This paper describes the specific extensions of the Pheno-App 2.0 for use in genebanks. The application is compared and discussed with two scoring applications known in the field of plant research, and an outlook on the further development of the Pheno-App is provided.

## 2 Related works

A wide range of applications are available for the mobile recording of phenotypic data. The Field Book and GridScore applications are briefly presented here as examples, as they are among the best-known applications in the field of plant research. The GBIS/B phenotyping application was used at the IPK genebank from 2006 to 2022 for recording phenotypic data. Many genebank-specific requirements were already implemented during the development of this application. During the period of use, experience was gained with both the software and the hardware used, which is taken into account in the evaluation of current scoring applications.

**Figure 1:**
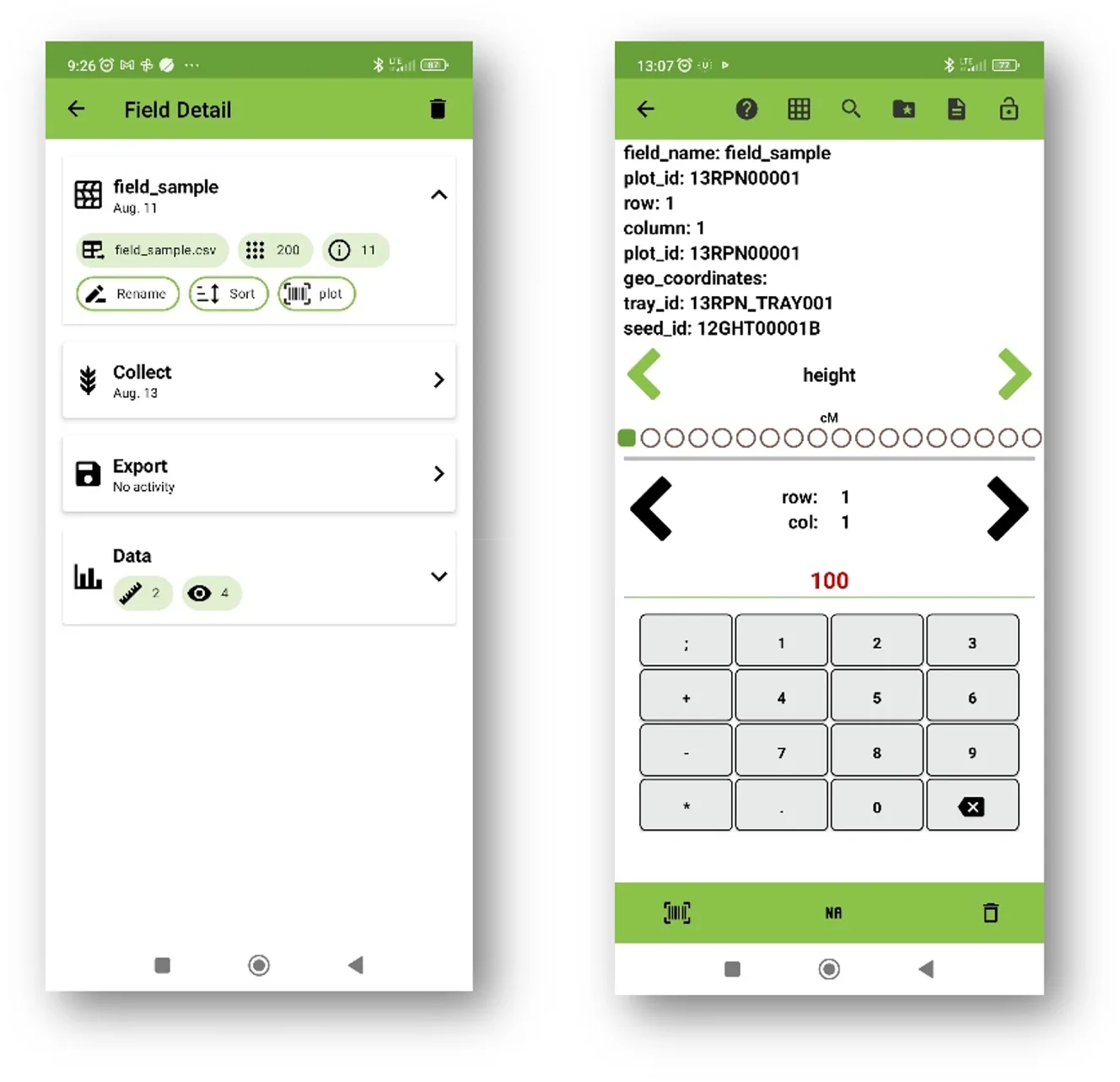
Field Book experiment overview (left), data input (right).

Field Book is an Android-based application available free of charge that was developed specifically for tool-supported data recording in breeding programmes [9]. The Field Book application interface is very minimalistic and offers intuitive data entry options for different data types thanks to special UI elements (see Figure 1). Furthermore, diagrams of the data already recorded in the experiment for the individual traits are available and the recording status is easily recognisable. All recorded values can be viewed and corrected in an overview. The Field Book application also supports barcode scanning and label printing on (mobile) Zebra printers.

**Figure 2:**
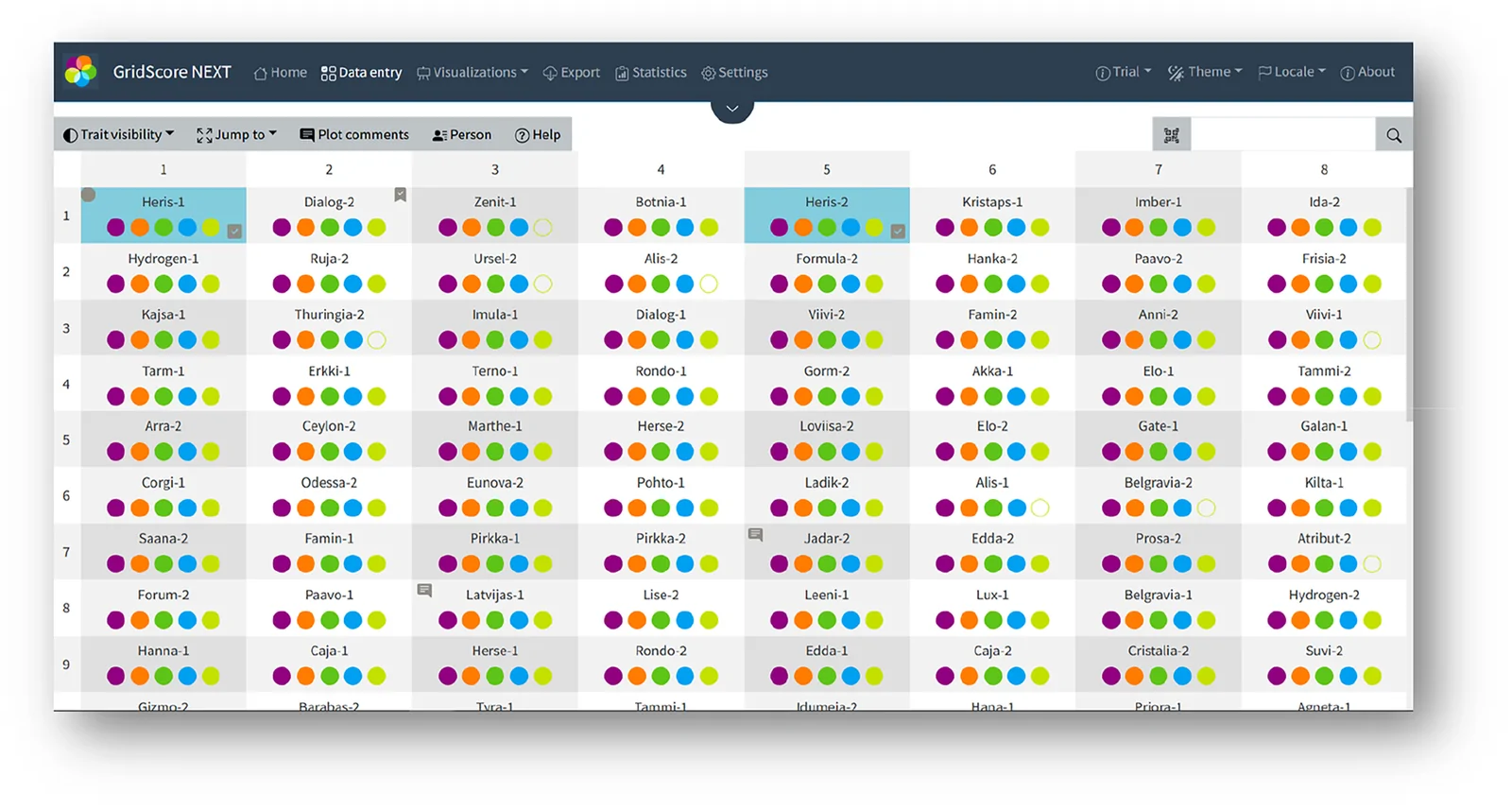
GridScore experiment overview.

**Figure 3:**
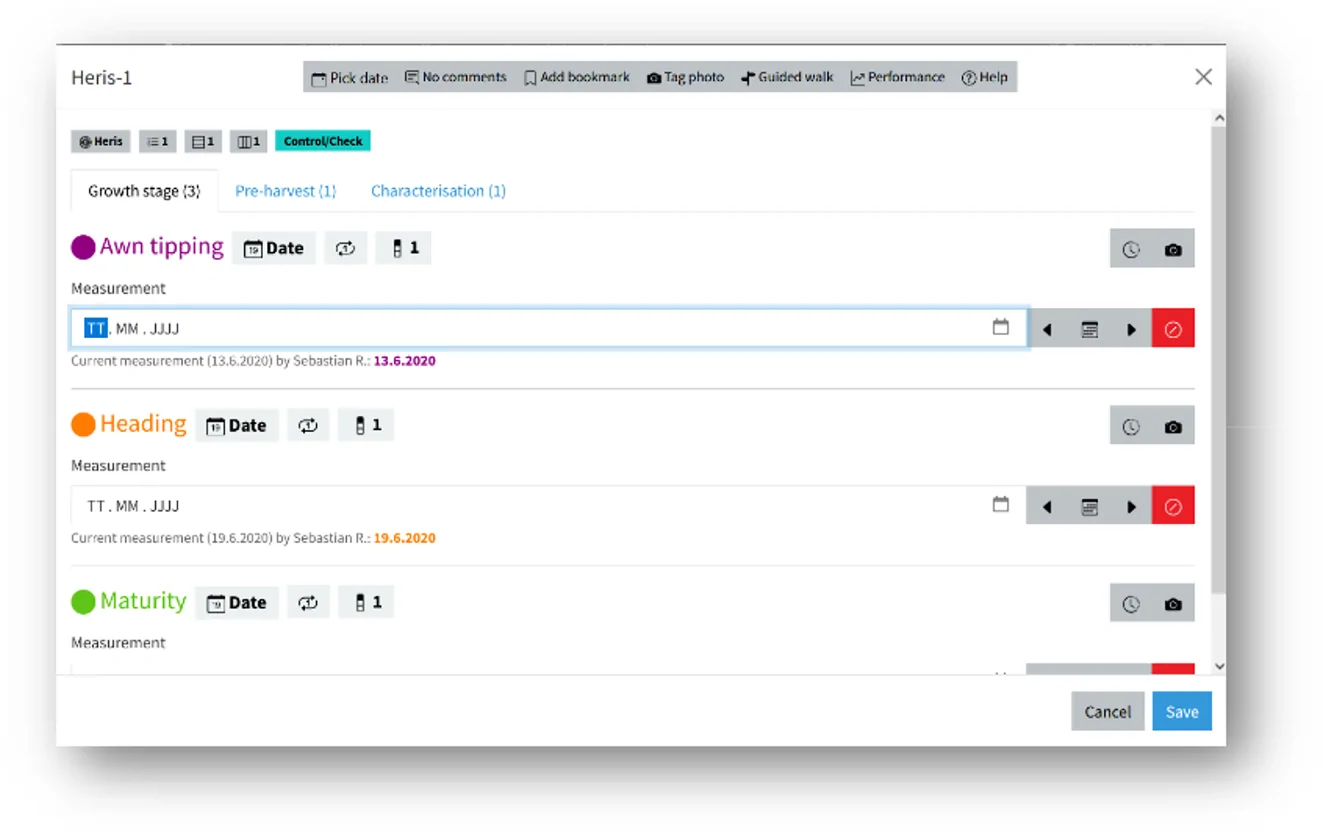
GridScore data recording.

GridScore is a web-based application designed for working with field plans (see Figure 2) [10]. It is developed at the James Hutton Institute in Scotland, and the current version, GridScore Next, is available free of charge as a web-based desktop version as well as an Android version. Traits for which a value has already been recorded are highlighted in colour in the field plan, so that the progress of the scoring is visible to the user at a glance. As well as Field Book, GridScore supports numerous data types. The application allows the definition of minimum and maximum values for numerical values and the number of value entries per trait. Comments can be saved for a plot. Figure 3 shows the dialogue for entering values for a demo experiment in GridScore Next: The name of the material and location information are displayed for the current plot. The data for the various traits can be entered below this, and the last recorded value is displayed below the input field. Various diagrams can be called up in GridScore for the recorded rating data, both for individual traits and for the entire experiment.

Compared to the two applications Field Book and GridScore, a scoring tool suitable for the use in genebanks needs to support more detailed recording of trait data: several values might have to be recorded for a trait, together with a percentage value and an optional comment to describe the observation and conditions. Comprehensive passport data needs to be displayed for each plot as well as reference values for the individual traits. Also a completion mode is needed, as for each location individually or for all locations uniformly, it should be possible to define which traits are to be recorded in full, without risking to forget a recording, while others might be explicitly neglected for some reason.

Current applications, such as Field Book and GridScore presented here, are very well suited for recording phenotypic data from homogeneous plant material, such as that used in research projects, due to their diverse functions and supported data types. When phenotyping heterogeneous material, more detailed input is necessary in order to describe the plant material completely and accurately. As an open-source application, version 1.0 of the Pheno-App provided a good basis for further developing the application for use in genebanks. The Pheno-App has already been used in projects at the IPK genebank, where positive user experiences with the application have been gathered. The following section explains the further development of the application to version 2.0.

## 3 Implementation

In order to adapt the Pheno-App for use at the IPK genebank, requirements were first collected, which will be briefly outlined here. Subsequently, the system integration into the existing infrastructure of the genebank will be presented. Furthermore, the new features of Pheno-App 2.0 compared to the previous version will be explained.

### 3.1 Requirements

According to the main tasks of genebank work as are taxonomic determination, documentation and regeneration of PGRs, specific requirements are placed on a scoring tool. Especially to perform the task of documentation and characterisation of heterogeneous material, but also to provide good quality of the data, the following requirements have to be met:

– Traits may have one ore many values with proportions to describe heterogeneous PGRs, like populations, landraces or crop-wild relatives in detail. Therefore, additionally to the trait value itself, an input element for recording the proportion needs to be present. It must be possible to add multiple values for one trait.
– A note may be optionally added to an observation, to describe the expression of a trait or conditions of the observation in detail.
– Adding notes describing the plot or general characteristics, should be possible.
– Displaying detailed information on traits:
  – Description of the trait, to have detailed information on hand how to observe a certain trait.
  – Descriptive image of possible trait values to help the user scoring the trait.
  – Reference values, which means the oldest scored values for a trait, together with proportions, for comparison with actual observations.
– Definition of different trait sets within one experiment should be possible, to be able to score PGRs of different crops in one experiment.
– Mark traits that have to be completely scored for all plots of an experiment. When navigating to the next unscored value these traits have to be taken into account.

Version 1.0 of the Pheno-App provided a good basis for replacing the previous GBIS/B scoring software. It was already possible to save multiple values for a single trait, but without the option of specifying proportions and adding a note. The completion mode could also be used indirectly, marking traits and using the first-empty-button to navigate to the next unscored trait. As part of the development of version 2.0, the requirements described above were implemented and an additional interface to GBIS was created, allowing it to be fully integrated into the genebank management system.

### 3.2 New features of the Pheno-App 2.0

**Figure 4:**
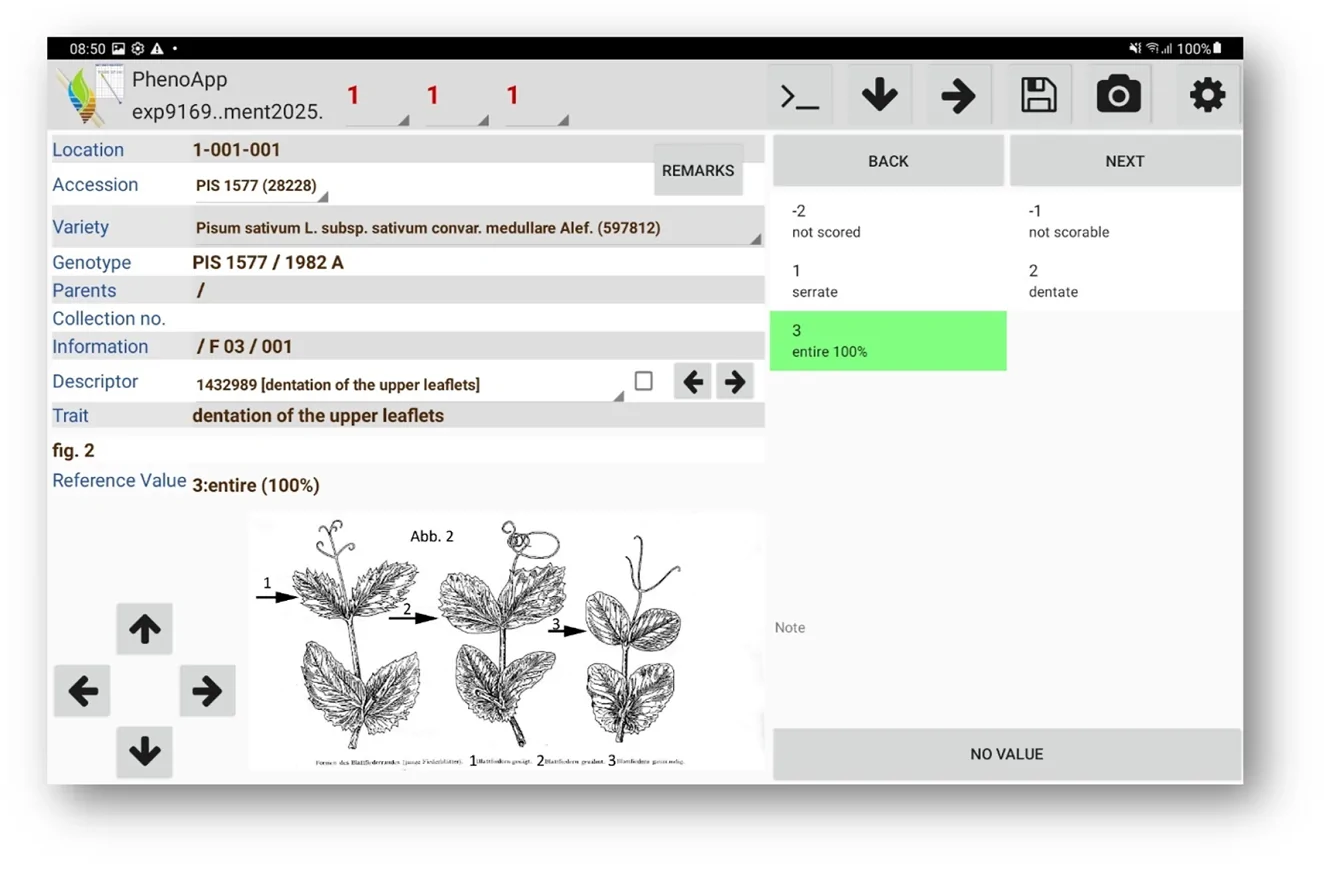
Main dialogue of the Pheno-App 2.0.

The Pheno-App’s main data entry window provides a clear overview of location information, the currently selected trait for scoring and corresponding input elements, as well as numerous options for navigating between the plots of the experiment and the traits https://discord.gg/bQePeeGh (Figure 4). A total of 10 pieces of information referred to the plot can be displayed. Furthermore, a description and an image are displayed for the currently selected trait, providing information on how a trait should be scored. Since a software keyboard is displayed for input, the available space can be optimally used for displaying information and input fields.

Plots can be selected directly via a three-part selection in the upper menu bar or by selecting the accession from the list. Navigation between the plots is possible by using the control pad at the bottom left of the screen or the ‘Next’ and ‘Back’ buttons along the current direction of the path long the plots. The Pheno-App currently supports two languages: German and English.

The layout of the interface has been slightly changed with version 2.0 compared to version 1.0, to display additional information for traits and scored values. A reference value is now displayed in the left-hand area of the location information, and several input elements have been added to the right-hand area, depending on the data type of the selected feature, to enable recording the proportion value and note and also to display multiple scored values for a trait.

The main changes to the application relate to modified functionality, such as navigation, and associated changes to the internal data model, which will be described in the following sections.

#### 3.2.1 Support of trait sets

**Figure 5:**
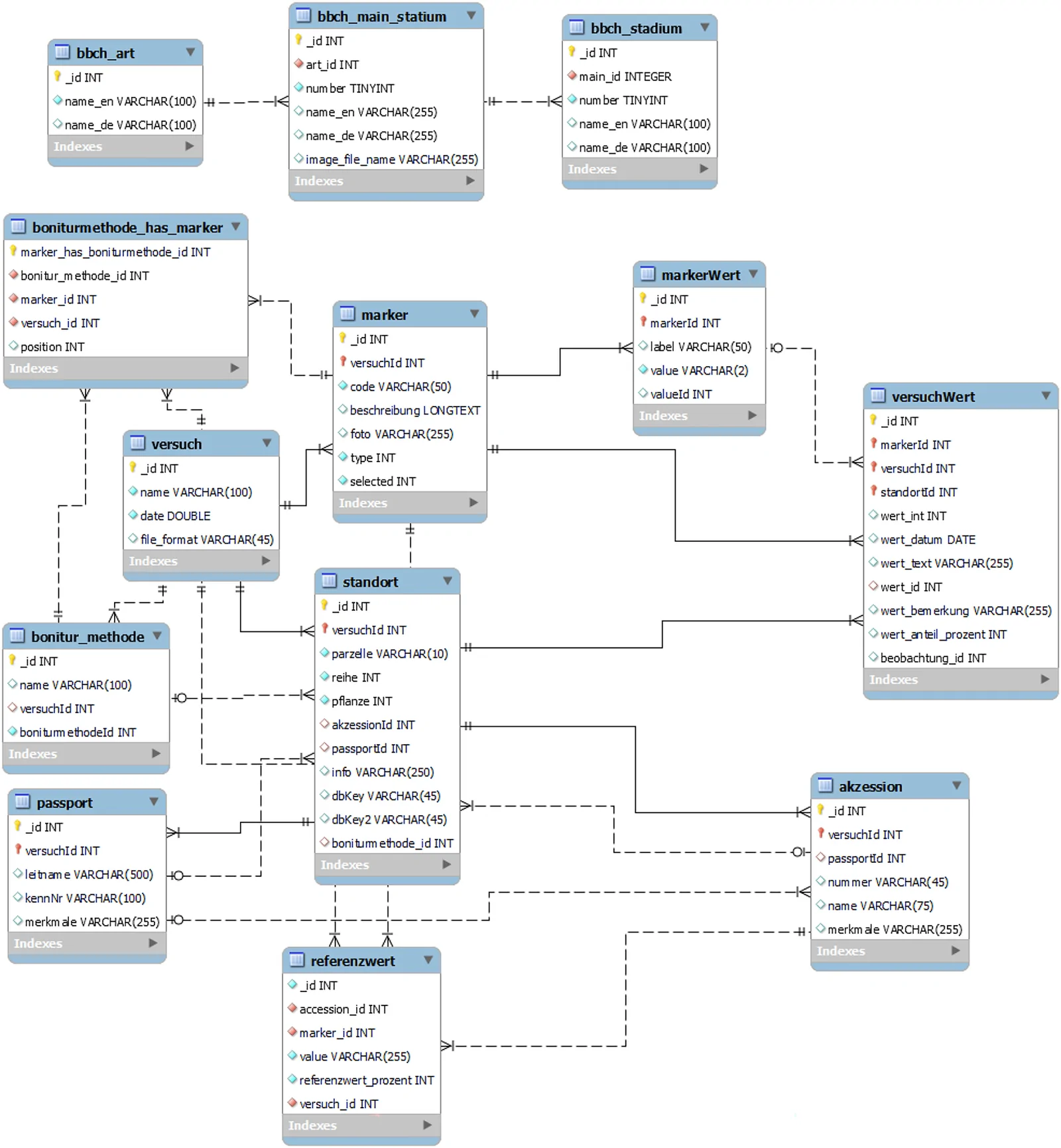
SQLite database schema.

For different crops, sets of traits with associated value ranges or value lists are defined and grouped into descriptors. In GBIS, descriptors are defined for crops. In order to transfer these descriptors to the Pheno-App and display the correct set of traits for a plot, the data model of the internal SQLite database had to be adapted. Figure 5 shows this data model: Each plot is assigned a descriptor, which in turn is assigned a set of traits via a link table. This allows traits to be reused in different descriptors for the same crop, e.g. to define descriptors of varying scope, or for different crops. The reuse of clearly defined traits, methods and value ranges in descriptor lists promotes the comparability of phenotypic data, not only between different genotypes, but also between different species or between cultivated and wild species. The Pheno-App offers the possibility to use such clearly defined descriptor lists.

When navigating through the plots of an experiment in the Pheno-App, only traits relevant to the respective plot are displayed to the user. Input and output routines of the application have been adapted to support multiple trait sets, depending on the given input file format.

Further adjustments to the data model include the assignment of reference values to traits and the extension of the scored value to include attributes for proportion, note and an optional identifier for the observation. The optional observation identifier can be used in conjunction with the observation date to control synchronisation with the target database.

#### 3.2.2 Recording multiple trait values with proportions and notes

**Figure 6:**
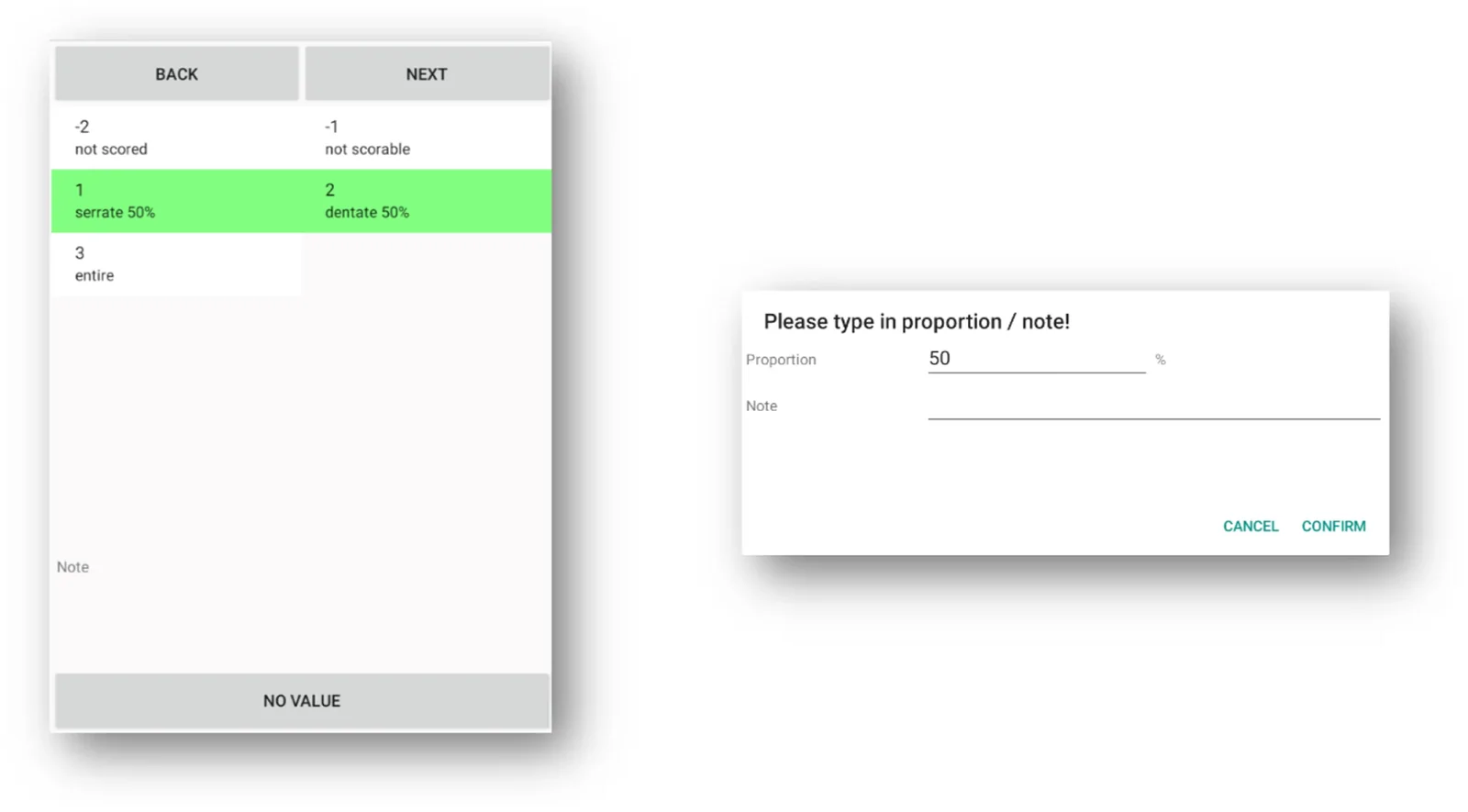
Category values select list and input mask.

**Figure 7:**
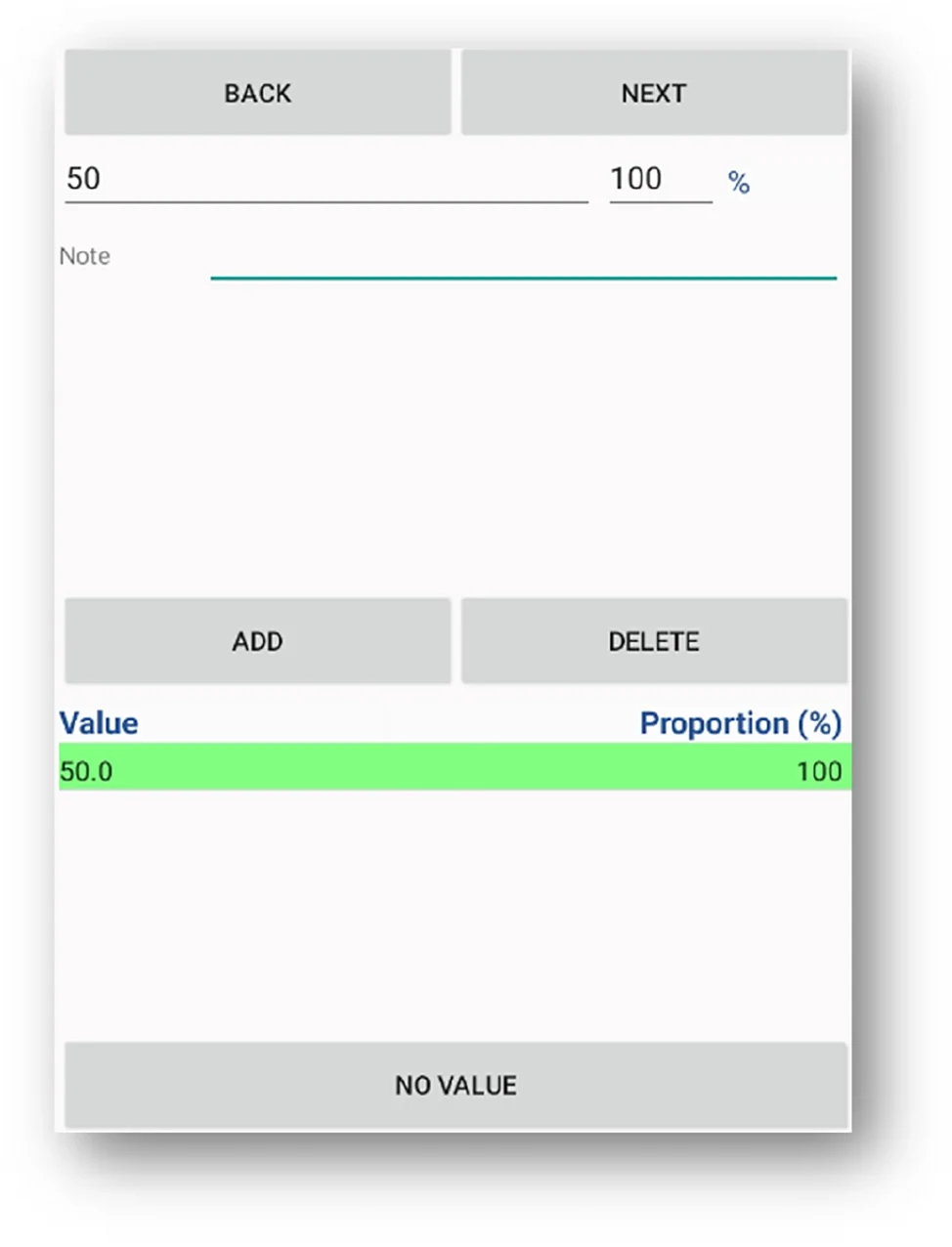
Input of number value with proportion and note.

The input elements for observation values have been supplemented with options for entering a proportion and a note for each value. For the category data type, proportions are calculated automatically when multiple values are selected (Figure 6). However, a proportion can also be entered explicitly. For all other data types, additional values must be added using a button (Figure 7). These are then displayed in a table below the input fields.

#### 3.2.3 Completion mode

The completion mode is useful for extensive experiments with many plots or many traits to be scored. Selected traits can thus be recorded for all plots without accidentally forgetting any entries. The Pheno-App offers the option of selecting traits for scoring. Together with the functionality of the ‘First-Empty’ button, you can navigate to the next plot where one of the selected traits has not yet been filled in. Unlike the Pheno-App in version 1.0, in version 2.0 the traits selection is permanently saved for the experiment in the internal database.

#### 3.2.4 Input- and output format

Compared to the previous version of the Pheno-App, the structure of the input and output files has also been changed. The input file consists of several spreadsheets, which have been expanded to include a spreadsheet for reference values and a spreadsheet for specifying the file format. The spreadsheet for plots information has been supplemented with additional optional columns containing identifiers for grow outs and the used descriptor. The spreadsheet for entering trait information has also been expanded to store the descriptors, the trait is assigned to. Furthermore, the enumeration of trait values for the category datatype has been supplemented by optional identifiers, as those are likely to be used in common database schemes. The ‘Reference Values’ spreadsheet contains information on the plot, accession, trait and reference value with an associated proportion to be displayed with a trait on a certain plot.

Since *descriptors* can be very extensive, the output format and the ‘Data’ spreadsheet for entering synchronised observation data were also converted from a column-based to a row-based structure. Furthermore, the implementation of the input and output routines was converted to a current version of the Apache POI libraries, so that the Excel format XLSX, which is common nowadays, is also supported. Since input files contain information about the format, older files from Pheno-App version 1.0 can still be loaded, and the output format is also generated accordingly using the old routines.

#### 3.2.5 Supported Android versions and compatibility issues

Due to support for the XLSX format and the necessary Apache POI libraries (version 5.2.3), backward compatibility with the Android SDK from level 26 onwards is guaranteed. This corresponds to Android version 8.0 (Android Oreo) and above up to Android version 14.

### 3.3 Integration into the genebank IT system environment

Although the Pheno-App can be used without a backend system, interfaces for connection to existing information systems were created at the IPK genebank to ensure easier handling for users.

Since 2005, GBIS has been used at the IPK genebank to manage data of preserved PGRs and support workflows. In addition, LIMS has been used since 2015 to manage phenotypic data for scientific experiments on external plant material as well as on accessions of the genebank collection. The Pheno-App has been used with LIMS in projects since 2022 [8]. In addition to passport and management data of the collection, the GBIS database also stores the phenotypic data collected whilst regeneration of the PGRs. Phenotypic data is thereby organised into experiments and linked to the accessions data. Once approved by a curator, it is also available to external users via the GBIS search and order portal.

An interface for exporting and importing experiment data between GBIS and the Pheno-App was developed as a web-based application in Oracle/APEX. It supports genebank users in preparing experiments and automates the generation of input files for the Pheno-App. All data necessary for carrying out the experiment, such as locations, accession passport data, descriptor lists and reference values, are extracted from the genebank management system database and are then available to the user within the Pheno-App.

In addition, a process has been established that links in the IPK LIMS system for importing and exporting the collected data both in and out of the Pheno App 2.0. IPK uses a commercial LIMS system that has been successfully established and continuously developed for over 10 years [11]. The system is a combination of an ELN for unstructured data and a RALIMS for structured data, organised hierarchically. It uses a relational ORACLE database management system, a connected hierarchical storage management system (HSM) for archiving LIMS-referenced primary data files and a Microsoft Windows Server cluster for hosting the RALIMS frontend. By using the same Oracle database, LIMS and GBIS can exchange data via SQL access, thus efficiently supporting the use of information from both systems.

## 4 Results and discussion

The Pheno-App 2.0 has been in use at the IPK genebank in connection with GBIS since 2023. It was initially tested in a two-year trial phase collecting user experiences and further improving functionality. By providing web-based interfaces to the internal management systems LIMS for project data and the GBIS for the genebank collection, the Pheno-App can be used with both systems. Furthermore, like its predecessor, the Pheno-App 2.0 can be used independently of a management system, as table-based files (XLSX, CSV) continue to be used as the input and output format.

Unlike other scoring applications, the Pheno-App 2.0 supports more detailed recording of phenotypic data by allowing multiple values to be recorded for a single trait with proportions and additional notes. This means that populations in which different expressions of a trait might occur simultaneously can also be fully described. It also provides useful information, like descriptor pictures and reference values of traits, to enhance scoring of PGRs.

Table 1 provides a comprehensive comparison of the three applications Field Book, GridScore and the Pheno-App 2.0.

**Table 1:** Comparing the phenotyping tools Field Book, GridScore and Pheno-App 2.0.

	Field Book	GridScore	Pheno-App 2.0
Experiment creation	Column- and row-based field layout directly in the application or file import from local storage/cloud, features defined identically for all experiments (can be activated/deactivated)	Experiments based on a field plan are created directly in the application and can be shared (cloud), additional information: experiment description, geocoordinates, visual markers, groups, people with roles, characteristics are defined per experiment.	Directly in Excel or other spreadsheet software or your own web-based interface (LIMS/GBIS), input files are stored in the local device memory, features defined per experiment and can be activated/deactivated
Input-/output-format	Direct communication with the database (BrAPI) or file-based (CSV)	File-based: FieldBook, Germinate;BrAPI	File-based, input: XLS, XLSX, output: XLS, XLSX, CSV
Supported data types/input elements	Numeric (with specific UI for percent, counter, range, angle), text, date, truth value, category, diseases (score), audio, geodata (GNSS, latitude/longitude), GoPro, photo, Zebra label	Numeric (with specific UI for range), text, date, Boolean value, category, geodata (latitude/longitude), photo	Numeric, Text, Date, Category, Photo
Layout/presentation of information	Maximum 16 pieces of information about the location; at least location identifier, row, column; optional plot	Maximum 6 pieces of information about the location; minimum location identifier	Maximum 10 pieces of information about the location; minimum location (plot, row, plant), identifier; 4 pieces of information on the trait, additional picture
Navigation	Row (2 directions); row, column, plot directly selectable (input), barcode scan;Guided Walk: rows forward/backward or zigzag	Row, column (3 directions); plot can be selected directly via field plan, barcode scan, or geocoordinate;Guided Walk (16 patterns): snake or zigzag	Row, column (2 directions); plot, row, plant directly selectable;Guided Walk (4 patterns) along rows, zigzag, walking direction can be changed at any time
Additional functionality	Search for data, e.g.: characteristics, location identifiers; overview display with correction option; input lock; cyclical recording; tutorial mode; statistics on the experiment and characteristics; completion mode;12 languages	Overview display with correction option; cycle recording; comprehensive statistics (experiments, personal evaluation, map display, evaluation of feature recording with timeline, heat map, charts for individual features (box plot, bar chart));3 languages	Additional information about the location can be recorded; BBCH stages (cereals, rice, corn, rapeseed, grapevine) Completion mode;2 languages (German, English)

The quality of phenotypic data can be significantly improved through tool-supported recording compared to recording on paper or with spreadsheet-based software such as MS Excel. The Pheno-App 2.0 is a tool that, unlike other existing mobile applications, has been well adapted to the working methods used in regeneration at genebanks. Since 2022, the IPK genebank has collected phenotypic data from 184 different crops in 93 experiments with a total of about 85,000 observations using the Pheno-App 2.0 for accessions managed through the GBIS system and the tool has been well accepted by the users. Their feedback has been very positive so far. Users rate the collection of phenotypic data both for project-related experiments and in the context of the regeneration of genebank accessions highly. They describe data collection with the app as intuitive and time-saving. The results of structured feedback rounds will be increasingly incorporated into plans for further development in the future.

## 5 Conclusions and outlook

With its current range of functions, the Pheno-App 2.0 is only inferior to other tools in a few respects, such as support for common interfaces, exchange formats or statistical evaluations. However, it supports more detailed documentation of phenotypic traits of PGRs and is therefore well adopted for use in genebanks, especially for the characterisation of heterogenous material such as populations or landraces. As a first step, all important requirements of genebank documentation work have been implemented and the Pheno-App has been tested in the field. Following an evaluation by users, the next step of the Pheno-Apps development will be the implementation of selected functions that further support data collection, such as the integration of barcode recognition software. Functions that further support the documentation of work during regeneration through to harvest are also to be implemented, such as the cleaning up of accessions or the collection of herbarium specimens. The connection of mobile label printers would also be conceivable. The possibility of documenting the co-occurrence of specific trait expressions also represents a possible functional extension that could enable a deeper analysis of populations, landraces or crop-wild relatives. To close the gap to other phenotyping tools, common exchange formats and interfaces will also be implemented. Machine learning-based technologies for image analysis could be used to automatically recognise certain traits and thus support phenotyping, such as the detection of disease infestation on plant parts.
